# Research, education, ethics consultation: evaluating a Bioethics Unit in an Oncological Research Hospital

**DOI:** 10.1186/s12910-022-00863-z

**Published:** 2022-12-09

**Authors:** Ludovica De Panfilis, Morten Magelssen, Massimo Costantini, Luca Ghirotto, Giovanna Artioli, Elena Turola, Marta Perin

**Affiliations:** 1 Bioethics Unit, Azienda USL-IRCCS di Reggio Emilia, Reggio Emilia, Italy; 2 Centre for Medical Ethics, Institute of Health and Society, University of Oslo, Oslo, Norway; 3 Scientific Directorate, Azienda USL-IRCCS di Reggio Emilia, Reggio Emilia, Italy; 4 Qualitative Research Unit, Azienda USL-IRCCS di Reggio Emilia, Reggio Emilia, Italy; 5 Department of Medicine and Surgery, University of Parma, Parma, Italy; 6 PhD Program in Clinical and Experimental Medicine, University of Modena and Reggio Emilia, Modena, Italy

**Keywords:** Ethics, Empirical bioethics, Clinical ethics, Ethics support services, Mixed-method research

## Abstract

**Background:**

This study aims to quantitatively and qualitatively evaluate the activities of a Bioethics Unit (BU) 5 years since its implementation (2016–2020). The BU is a research unit providing empirical research on ethical issues related to clinical practice, clinical ethics consultation, and ethical education for health care professionals (HPS).

**Methods:**

We performed an explanatory, sequential, mixed-method, observational study, using the subsequent qualitative data to explain the initial quantitative findings. Quantitative data were collected from an internal database and analyzed by descriptive analysis. Qualitative evaluation was performed by semi-structured interviews with 18 HPs who were differently involved in the BU’s activities and analyzed by framework analysis.

**Results:**

Quantitative results showed an extensive increment of the number of BU research projects over the years and the number of work collaborations with other units and wards. Qualitative findings revealed four main themes, concerning: 1. the reasons for contacting the BU and the type of collaboration; 2. the role of the bioethicist; 3. the impact of BU activities on HPs, in terms of developing deeper and more mature thinking; 4. the need to extend ethics support to other settings. Overall, our results showed that performing both empirical bioethics research and more traditional clinical ethics activities at the same unit would produce an impetus to increase collaboration and spread an 'ethical culture' among local HPs.

**Conclusions:**

Our findings contribute to a growing body of literature on the models of clinical ethics support services and the role of empirical research in bioethics internationally. They also prepare the ground for the implementation of a multidisciplinary Clinical Ethics Committee (CEC) that aims to support the BU’s ethics consultation service within the local context.

## Introduction

Complicated health care decisions often involve conflicts of values. It is not unusual for patients and their families to seek advice about ethical issues from their clinicians, who in turn may strive to provide informed and well-reasoned responses to such requests [1].

To satisfy the needs of patients to receive more comprehensive and personalized ethical care, further support targeting health care professionals (HPs) and patients have been required [2]. The last several decades have seen increasing development and implementation of services specialized in dealing with and promoting the ethical dimension of clinical practice through clinical ethics support services (CESSs) [3, 4].

The increasing engagement with CESSs and clinical ethics leads to a growing focus on empirical research in bioethics. Indeed, the increase in empirical methods of research in bioethics can contribute to the integration of ethics consultants into the clinical setting [5]. Research activity can play an essential role in ethical clarification and decision-making because it can translate abstract principles into workable practices. It can also ensure that bioethicists are in touch with the actual experiences of those affected [6].

As we reported in a previous study [7–9], in Italy no national legislation exists about CESS’s role and functions. However, some local, unregulated and spontaneous experiences of CESSs has been implemented across different regions, especially in the North of Italy [10–13]. The National Committee for bioethics (CNB) issued an opinion in 2017 on the urgency of implementing clinical ethics committees throughout the country [14]. Moreover, in 2021 CNB published a statement about the role and competencies of the “expert in bioethics”/ethicist [15]. Also, the current debate around medical assistance in dying, following some leading cases, has renewed attention on the CESS’s functions [16].

The Bioethics Unit (BU) in this study is a research unit implemented as a pilot project by the Scientific Directorate of the Local Health Authority AUSL-IRCCS of Reggio Emilia. It is a local health service consisting of six hospitals and 6 districts encompassing the 42 communes of the province, and provides services on a total area of 2.291 Kmq. The BU was established in 2016 inside one of the hospitals, the Oncological Research Hospital (ORH), a 900-bed service accredited as a Comprehensive Clinical Cancer Institute (OECI). Since its implementation, the aim of the BU was to promote evidence-based ethics through research activities, ethics consultation and clinical ethics, and training programs. In other words, the mission of the BU is to promote a ‘bedside’ form of ethics, where the concrete experience of the health care relationship represents the starting point for improving quality of care [17] and fostering a personalized approach by enhancing HPs’ ethical competences. The BU works within the ‘empirical turn in bioethics’ framework. Empirical bioethics is an interdisciplinary research area that integrates empirical, qualitative, and scientific analysis with ethical analysis. The aim is to stimulate the translation of general moral principles into concrete and specific action-driven guidelines that are morally justified and workable in practice. [6, 18, 19].

Specifically, the BU’s activities are divided into four main areas: research related to ethical issues of clinical practice employing qualitative and quantitative methods; ethics consultationfor individual HPs; ethics supervision for the health care team; and educational programs and training on the ethical aspects of care for HPs. These activities are not necessary related, but they often run simoultaneously. A description of the BU’s activities is provided in Table 1.

**Table 1:** Description of the Bioethics Unit’s activities and aims

Activity	Promoted by	Dedicated to	Description of the activity	Aim
RESEARCH	All the BU members or other services and wards	Other services and wards	Development, implementation and evaluation of research projects related to ethical issues of clinical practice, employing qualitative and quantitative research	To promote ethical reflections and knowledge among HPs, especially regarding the following topics: patient engagement and shared decision-making, advance care planning (ACP), Advance Directives (AD), end-of-life issues and palliative care, pediatric palliative care, and ethics of resource allocation
ETHICS CONSULTATION	Only the Head of the BU	Individual HPs or healthcare teams, especially in urgent circumstances	Provision of retrospective and prospective ethics consultation by a single, face-to-face consultation	To support HPs in resolving ethical conflicts or deal with the complex decision-making process
ETHICS SUPERVISION	Only the Head of the BU	Healthcare teams, during their regular meetings	Provision of structured ethical supervision during the care team meetings	If ethical aspects emerge, the Head of the BU participates in the discussion, supporting HPs to focus on the ethical dimension of care and deal with emerging moral complexities
EDUCATION AND TRAINING	All the BU members	Single wards on a specific request, or all HPs	Implementation and provision of training programs and ethics education by face-to-face and experiential classes	To promote ethical reflections and knowledge among HPs, especially regarding the following topics: patient engagement and shared decision-making, advance care planning (ACP), Advance Directives (AD), end-of-life issues and palliative care, pediatric palliative care, and ethics of resource allocation

The research projects promoted by the BU are dedicated to developing, implementing, and evaluating services and tools related to ethical issues of clinical practice. They employ qualitative and quantitative methods. Some examples are those pertaining to advance care planning tools, advance directives implementation, ethics training evaluation, and development of ethical skills.

Ethics consultation is on demand and dedicated to individual HPs or medical teams, especially in urgent circumstances.

Ethcis supervision is organized monthly and it is dedicate to the healthcare team. The bioethicist participates in the team meetings and if ethical aspects emerge, the Head of the BU engages in the discussion.

Ethics education and training promote ethical reflections and knowledge among HPs. They are organized following HPs request or promoted by the BU. They regard especially the following topics: patient engagement and shared decision-making, advance care planning (ACP), Advance Directives (AD), end-of-life issues and palliative care, pediatric palliative care, and ethics of resource allocation.

Currently, the BU is composed of one employed senior researcher, one PhD student, and two research consultants involved in the BU activities in different ways. The BU also promotes and manage two institutional clinical ethics services: a Clinical Ethics Committee composed by 15 members [20] and an in-hospital service to inform people about end-of-life rights and advance directives. The BU team leader oversees the running of the service and meets with the Scientific Directorate management. As it is, the BU represents a CESS with a specific focus on research activity.

According to the literature, the evaluation of ethical interventions in clinical practice helps understand (a) user satisfaction, (b) penetration of the services among HPs, and (c) the impact of the service on the care provided, as well as for collecting useful data to improve the quality of the services provided [21]. The goal of this study is twofold:

- to quantitatively describe the BU activities carried out in the five years since its implementation (2016–2020);
- to understand the HPs’ perception of the BU activities and their perceived impact on clinical practice.

## Methods

This is a mixed-method study that followed a quantitatively driven and explanatory design [22]. Subsequent qualitative data build on the initial quantitative findings with the aim of expanding on them and offering a more in-depth understanding of the research questions through users’ perceptions.

### Quantitative study

#### Data collection

Quantitative data on the BU’s activities from January 2016 to December 2020 were collected to describe the activities in terms of hours/time spent, collaborators, and topics covered.

Information on research projects promoted and implemented by the BU, the number and length of ethics consultations, educational programs or training, the topics covered, and the units and institutions that collaborated in these activities were collected from an internal database.

#### Data analysis

The quantitative data were analyzed by descriptive statistics using IBM SPSS Statistics 26, by an expert researcher in biomedical statistics (ET). Two databases were generated, one for research activities and one for educational programs or training, consultation, and supervision activities. The datasets were analyzed independently except for the data related to the topics addressed.

### Qualitative study

#### Population and setting

We performed a purposive sampling varying eligible participants’ characteristics. Participants were identified among units that have differently collaborated with the BU, accordingly with emerging quantitative finding.

Participants had to meet the following inclusion criteria:

- They were working at the local Health Care Service of Reggio Emilia.
- They had been involved in at least one of the activities provided by the BU between 2016 and 2020.

#### Data collection

A series of one-to-one qualitative interviews were conducted to understand the perceptions of HPs who collaborated in activities organized by the service. The interviews focused on the BU activities and their perceived impact on clinical practice. Due to the restrictions imposed by the pandemic, all the interviews were conducted online except for one, which was held at the participant’s office. Two researchers (LDP and MP) developed the semi-structured interview topic guide. LDP is the Head of the BU, while MP is a PhD student in Clinical and Experimental Medicine. They both work at the BU and have a background in qualitative research. The interview guide was revised by a third researcher (LG) who is the head of the qualitative research unit. The main topics were (a) the participants' evaluation of their involvement with the BU in terms of satisfaction and the impact on their clinical practice; (b) further needs regarding ethical aspects of care; (c) expectations for future collaboration with the BU. The interview guide and related exemplifying questions are reported in Table 2.

**Table 2:** Interview topic guide

Theme	Exemplifying questions
*Evaluation of the participant’s collaboration with the BU in terms of: satisfaction, impact on clinical practice and within the health care relationship*	-Can you tell me what the activities of the BU are? And what are the aims of the activities promoted by the BU? -Which of these activities have you been involved in? What was the reason for your involvement? -What do you think about the activities you took part in? -How would you evaluate the impact of this experience on your clinical practice? And on the care process? (For example, in the relationship between medical teams or different settings?) -Considering your personal experience, do you remember a particular situation when the BU was particularly significant? Can you describe it with an example?
*Further needs regarding ethical aspects of care*	- Considering your work and the BU activities, are there other topics that need to be examined more in depth? If so, can you give an example? -Considering the place where you work and your colleagues and medical teams, are there other situations that need to be examined more in depth? If so, can you give an example?
*Expectations on future collaboration with the BU*	-The BU is thinking of expanding the ethics consultation service. What is your opinion about this? -What activities do you think the BU should promote in the future?

A letter was sent to each respondent to invite them for an interview at a convenient date and time. The interviews were audio-recorded with the participants’ consent. All the interviews were conducted by three researchers (MP, GA, and CG) trained in qualitative research. No repeat interviews were conducted, and the transcripts were not returned to the participants for comments or corrections.

### Data analysis

The semi-structured interviews have been transcribed verbatim. We followed the thematic framework [23]. It was developed by MP and GA, and supervised by LG, combining a deductive approach with an inductive one: themes were initially pre-selected based on the research questions (deductive approach), then themes were generated from the data though open coding (inductive approach). Firstly, MP and GA read the transcript of two interviews, writing down first impressions and notes. Then, they coded the texts, and analyzed themes independently before reaching inter-coder agreement. Using the agreed-upon themes, MP analyzed each interview by applying the framework to make recurrent themes emerge. GA supervised the work. Subsequently, the two researchers discussed and agreed on emergent themes and subthemes. LDP, LG, MM and MC contributed to analysis and the presentation of the final results.

## Results

### Quantitative results

**Fig. 1:**
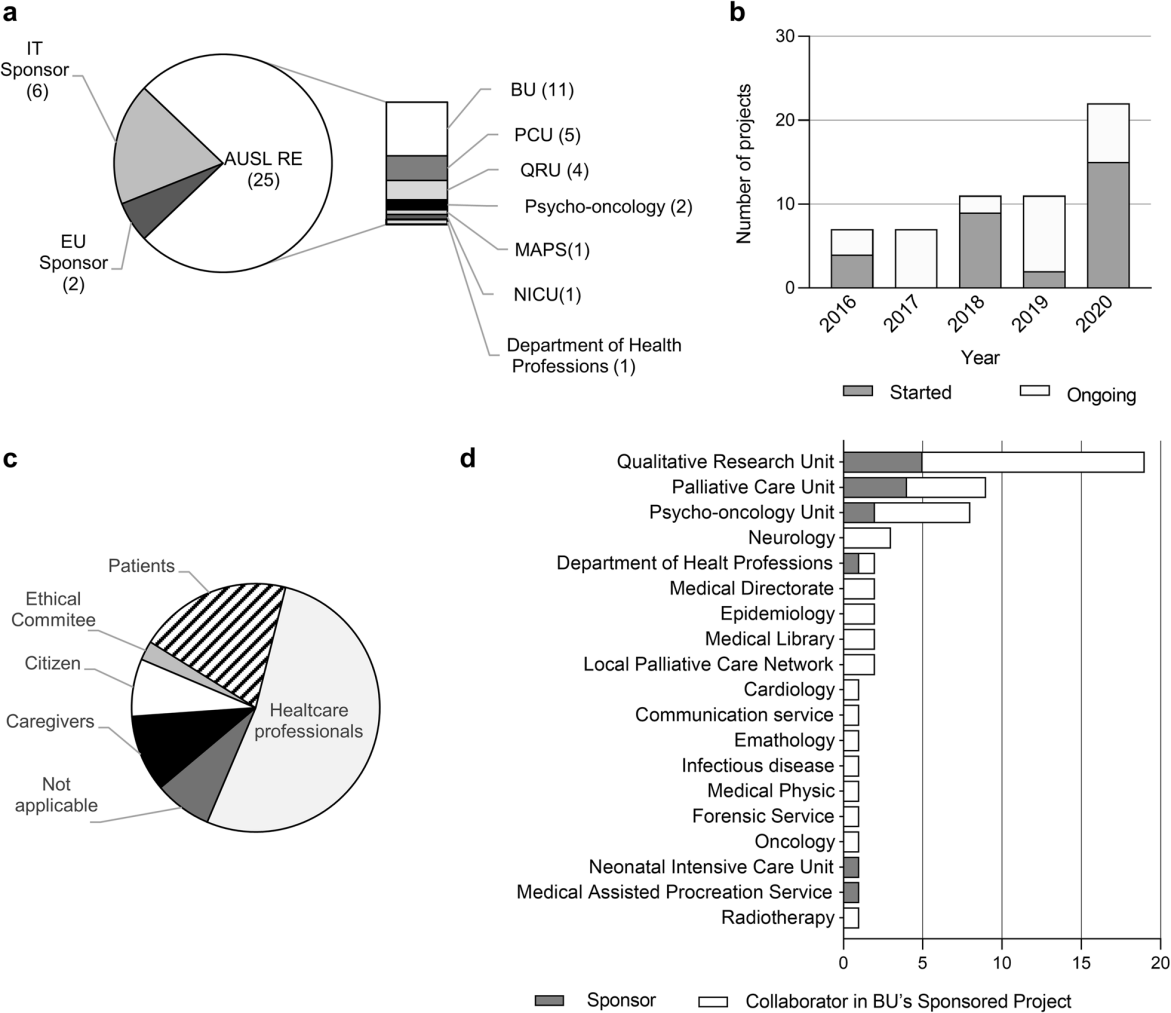
BU’s research activities. Quantitative analysis of the number of projects, collaborators, and target audience of BU’research activities from January 2016 to December 2020. **a** Sponsor of the research project in which BU is involved. **b** Initiated and ongoing project per year. **c** Research projects target audience. **d** Units that have collaborated with the BU either as sponsors or collaborators in BU-sponsored projects. *IT* Italian, *EU* European, *AUSL-RE* Azienda AUSL IRCCS di Reggio Emilia, *PCU* palliative care unit, *QRU* qualitative research unit, *MAPS* medically assisted procreation service, *NICU* neonatal intensive care unit

From January 2016 to December 2020, the BU participated in 33 clinical and organizational research projects. Most of them were promoted by the Azienda USL IRCCS of Reggio Emilia structure (n = 25), while 8 projects were led by other Italian institutions and European organizations (n = 6 and n = 2, respectively). The BU directly promoted 11 research projects (Fig. 1a).

The number of research projects in which the BU was involved increased over the years and reached its maximum in 2020, when 15 new research projects were initiated while 7 were still ongoing (Fig. 1b). The research projects had several target audiences, including patients, caregivers, citizens, and ethics committees. However, more than half were designed for healthcare professionals (63.6%) (Fig. 1c). Among the 33 research projects in which the BU collaborated, some consisted of updating perspectives on roles and practices in specific settings to revise or produce new care pathways or implement new services. Others aimed to explore or comprehend whether and how healthcare professionals make sense of and handle ethical issues in clinical practice. Some research projects aimed to assess the effectiveness of a structured ethical intervention (e.g., Advance Care Planning) for a target population. All of them have as their main outcomes improving quality of care and quality of assistance and scholarly papers published in peer-review journals. The BU’s research activity was carried out in collaboration with 19 units/services, both involved and not involved in patient care (n = 11 and n = 8, respectively). Ten units collaborated with the BU on only one project, 6 units collaborated on two or three projects, and 3 units were involved in more than 8 projects. Four services collaborated both as sponsors and collaborators in projects sponsored by the BU (Fig. 1d).

**Fig. 2:**
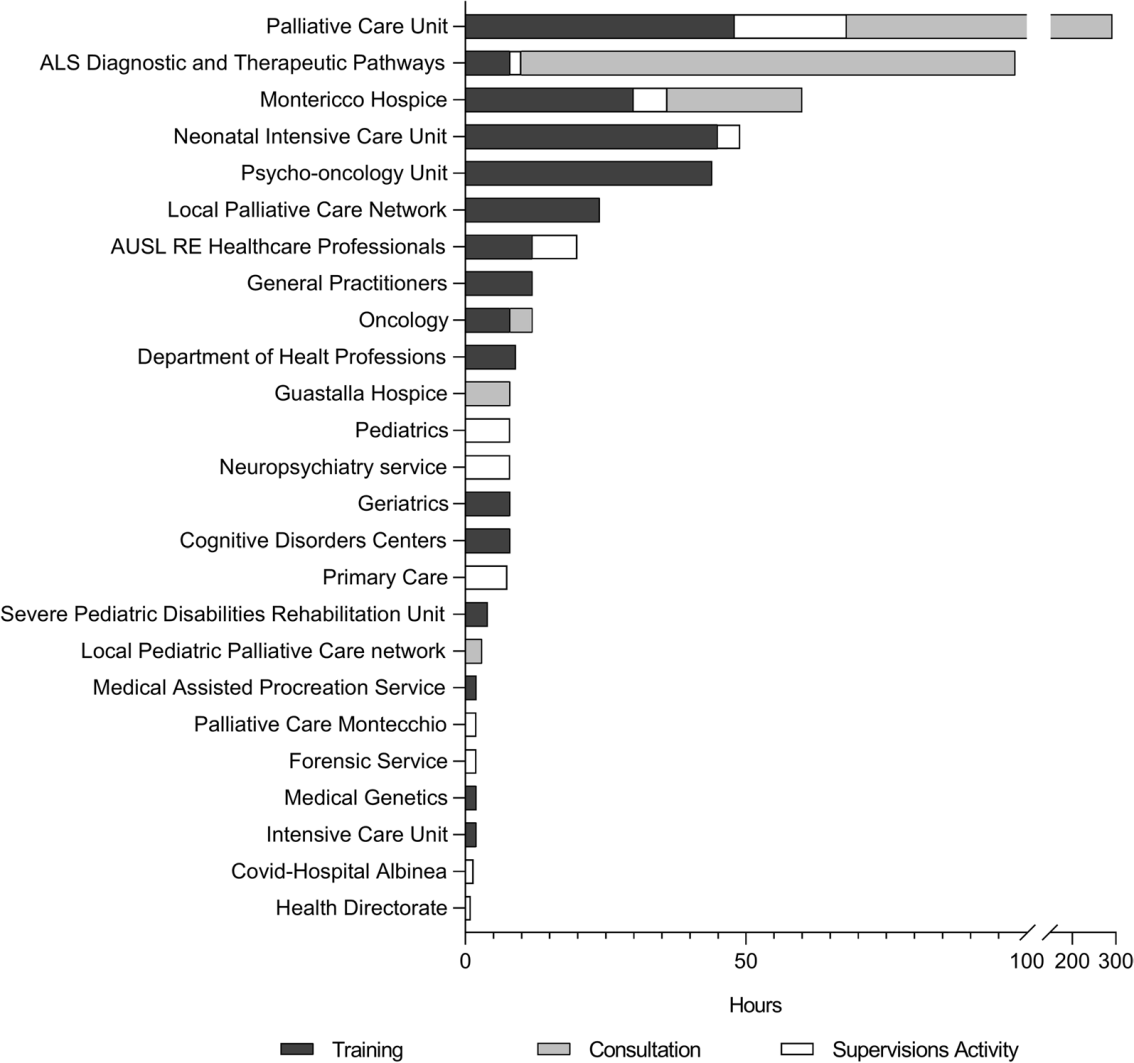
BU’s consultations, training, and supervision activities. Hours spent from other structure in AUSL-Re BU consultations, training, and supervision activity

Consultation, training, and supervision activities involved 25 services/units, 22 of them directly involving patient care, while three were not clinical services (health directorate, health professions directorate, forensic medicine). Ten units were engaged in community health service, while 15 were hospital ones (Fig. 2). The number of hours spent by the services on activities led by the BU varied widely, ranging from 1 h of the health directorate to 292 h of the palliative care unit (median: 8; IQR 2–20).

The majority of services/units collaborated in one of the 4 areas of BU activity (n = 25), 10 services/units collaborated in 2 or 3 areas (n = 6 and n = 4). Only one unit, the Palliative Care Unit (PCU), collaborated in all the 4 main areas of activity.

**Fig. 3:**
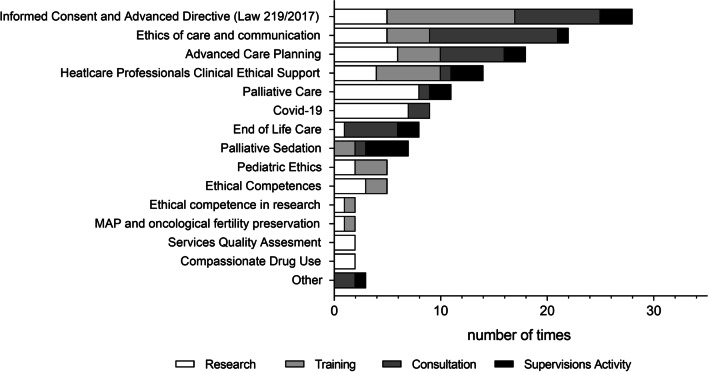
Topics addressed in BU’s activities. The graph showed the number of times each topic was the subject of one of the activities conducted by the Bioethics Unit

Finally, we looked at the topics addressed in the BU’s activities. Most of them were addressed in more than one activity, and 4 were addressed in all the areas. All issues except the ethics of deep palliative sedation were addressed from a research perspective (Fig. 3).

### Qualitative results

An appropriate sample of participants were recruited among units that had collaborated with the BU. Starting from the results of the quantitative analysis, we identified the following variables of the units where the participants work:

- Time spent in research projects;
- Time spent in consultations, educational programs or training organized/led by the BU;
- The extent of the collaboration between the participating unit and the BU;
- The type of unit participating, in terms of hospital/local community; pediatric/adult service; clinical/non-clinical service.

The units were first divided into three groups based on the total time spent in research projects, ethics consultations and educational programs or training organized/led by the BU: low level of collaboration (≤ 2 h), medium level of collaboration (between 3 and 12 h); high level of collaboration (> 12 h). Within each group, we identified units that differ from the other variables. Finally, we identified participants from selected units.

We identified and contacted 22 participants. Of these, 4 did not reply, while the others showed a great deal of interest in the project. The final sample included 18 participants: 16 female and 2 male HPs. Two were psychologists, one researcher, one biologist, one physiotherapist, one speech therapist, six nurses and six physicians. The participant characteristics are summarized in Table 3.

**Table 3:** Participants characteristics and collaboration with the Bioethics Unit

Cod	Professions	Working Unit	Collaboration with BU (R, EC, ES, E&T)	Time of collaboration	Level of collaboration (high, good, medium, low)
01	Physician	Hospice	*EC, ES, E&T*	*2018–2020*	High collaboration
02	Physiotherapist	Rehabilitation Unit for Severe Childhood Disabilities	*Only E&T*	*2018, 2020*	Medium
03	Physician	Intensive care	*Only E&T*	*2018 and 2020*	Medium
04	Speech therapist	Paediatric care	*E&T and ES*	*2020*	Good
05	Phisician	Covid-hospital	Only EC (Covid)	2020	Low
06	Psychologist	Neuroloy	Only R (4 projects)	Since 2018	Low
07	Psycho-oncologist	Psycho-oncology Unit	*E&T and R (9 projects)*	*2015,2018,2020*	High
08	Methodologist	Qulitative Research Unit	Only R (18 projects)	Since 2016	Low
09	Physician	Palliative care Unit	*E&T, ES; EC, R (13 projects)*	*since 2016*	High
10	Nurse	Palliative care Unit	*E&T, ES; EC, R (13 projects)*	*since 2016*	High
11	Oncologist	Oncology	*E&T, ES; EC, R (1 projects)*	*2016, 2019- 2020*	High
12	Nurse	Neonatal intensive care	*E&T, EC, R (2 projects)*	*2017–2020*	High
13	Nurse	Hospice	*Only ES*	*Since 2020*	Good
14	Nurse	Hospice	*E&T, ES; EC*	*2018–2020*	High
15	Biologist	Mediaclly Assisted Procreation Service	*E&T, R (2 projects)*	*2018–2020*	Medium
16	Nurse	Hospice	*Only ES*	*Since 2020*	Good
17	Physician	Rehabilitation Unit for Severe Childhood Disabilities	*Only E&T*	*2018 and 2020*	Medium
18	Nurse	Neuropsychiatry	*Only EC*	*2019*	Good

For further description of each activities, please, see Table 1

R: Research activity; EC: Ethics consultation; ES: Ethics Supervision; E&T: ethics education and training

The interviews were performed between February and March 2021 and lasted an average of 31 min (range 15–55).

Framework analysis identified the following 4 main themes and 6 related subthemes. Table 4 presents the themes and sub themes and corroborates the analysis with selected quotations.

**Table 4:** The 4 key themes, related sub-themes and meaningful quotes

Theme	I	Meaningful quotations
1. Ways and Meanings of the BU’s collaborations	1.1 How and why to access the BU	So in certain circumstances, I asked myself: Which person is the most competent to help you with this specific question? And I autonomously identified her (*the Head of BU*)" (cod. 1.4). We certainly made the initial request, because we needed an opinion, a point of view different from ours. This, perhaps, is ethics: a different vision from ours. (C.15.10) The reasons that led us to contact the BU were: first, the fact that the bioethics unit exists. This is the main reason: it is not so common for a hospital to provide this resource (c.6)
	1.2 The BU’s role and activities	If we talk about the ethics training (…) it brought attention to topics that we usually lack some knowledge of (C 2.3) In my innermost thoughts, I thought (my choice) was right, but in that moment (…) I needed someone to rationalize all the choices and the path of care, someone who could support me (c.5.18). In the educational activity, we discussed specific cases, (…) which created a bit of disagreement within the care team regarding the decisions to be taken on palliative sedation (…), and this made it clear to me and to the nursing coordinator that there was a need to increase the training activity on this topic (c.11.1.5). I believe that the BU’s aim is very clear, which is to give us more confidence and provide ethical support, not only methodological, towards choices that have to made…choices that are often less clinical than those taken elsewhere. (We work in a unit where) non-clinical choices have equal dignity to clinical choices (C.16.3).
2. The role of the bioethicist and organizational aspects	2.1 Personal attitudes and specific competencies	The strongest point is the training in the field, namely the discussion of real cases together: then you understand the whole theoretical part (c.10) The first times I changed my point of view on the case, being less focused on ‘doing’, it was a shock to me too. Then… knowing that no one judges you… the bioethicist is the right figure for this type of support. (C.18.20) In our setting there are, as well as perhaps in other settings, cultural aspects that in my opinion it is important to be aware of, and from this point of view, having referents who specifically study these things, and have a background and the ability to follow the current ethics questions is fundamental for having feedback, also for the overall growth in the system of our department (c.11.41)
	2.2 Organizational aspects	If I have a symptom X, I do something to cure the symptom X, and I need to have the result immediately. On the contrary, considering the care path, I do something, and I will see the result after a long time, it is a path built in many steps. This is perhaps the difficulty of making the two facets interact with many other facets: I think this (*ethics*) is a facet of our work, but it doesn’t always fit in with the timing we are used to working with. (c.3.28) If there is only one person (the head of BU), you do not know who to refer to. Probably there is a limit (…) in the sense of having only one contact person. (c.11.45) I didn’t even know (the BU) existed, (…) I could never have made the contact if I hadn’t found out from someone else. (…) knowing that it exists is important, to spread this opportunity. (c.5.43) (*There are*) organizational (*limitations*) but also regarding cultural aspect of both executives and us as professionals: because we are inside this logic of doing and doing, and you are not allowed to think about the ethical aspect of doing…(reflecting on it) is experienced as a luxury (c.2.21)
3. Impact on the HPs’ attitudes	3.1 A deeper way of thinking	It is very important to have other ‘’two eyes’’ trained on this type of comparison. It allows you to refine, to mature, to give a little more depth to your assessments, to your orientations." (c.1.10) The possibility of drawing on a professional who is not strictly clinical but also has a wider philosophical tradition, which is more extended compared to the questions we are usually accustomed to thinking, but which in real life concern the daily choices of clinicians and patients (c.6) In the end, (the Head of BU) not only helps you to deal with the question that arose, but also helps to strengthen the group and therefore in my opinion it is a doubly positive result. (c.13.22) Then there were also situations in which we realized that the problem was not ethical but was more relational or organizational. But (the ethics intervention) was useful anyway, because we also understood what kind of problem it is: since we work within complexity, it helps a lot. (c.14.10)
	3.2. Identifying new questions in clinical practice	I think there’s been a positive impact, in the sense that we, as clinicians, are far ahead clinically. (…) She (the head of BU) suggested that we go step by step, take small steps, set small goals and see on the other side how the parents respond. It is not easy for a parent to become aware of the child’s illness. In this way, instead, they arrive prepared, but we must not do everything immediately. (C.18.9) Sometimes we clash also, we clash in the sense of a comparison among us, among our different points of view, and in my opinion the tools that are offered to us (by the BU) help the care team to be a little more at peace. (c.14.20) (There has been) an improvement in the relationship with patients. (…) While before, the meeting with the patients took place on two slightly separate levels, we at the top and patients at the bottom, I saw this distance was reduced. And so more and more professionals are trying to put understanding at the core of the health care relationship, even when things aren’t really great. (c.15.33)
4. Further needs		I think that considering all this no longer in an informal way but in a planned, structured way can be absolutely useful." (1.16) More structured, because when we need (the BU intervention) the situation is probably already too far advanced. If I had a consistent presence, (…) she makes me aware in time that there is a need for something more, to implement a care pathway where I usually arrive too late. (c.3.23) So sometimes I asked myself: Why not bring this way of training to other departments? Because an oncologic patient is in all departments, because the “questions of meaning” arise in all the departments (…) (c.13.38) What I think and hope is that having a system within the health care facility (dealing with ethical aspects of care) will push (…) all the departments to provide health care professionals with an ethical basis, because ethics is the basis of the profession and still too many nurses do not perceive that (…) goes beyond in so many ways (C.16.35) So I hope it really becomes a way to bring it to everyone, because everyone has ethical doubts but sometimes they don’t know them and so the crises arise. (…) I think this year has brought it to light a lot (due to the COVID-19 pandemic), (…) there have been many ethical choices and they often have not been perceived, and this perhaps has aggravated a crisis that was already serious from a clinical point of view. But the clinical practice raises questions that are easier to solve than the ethical ones, and so I think it’s right to care for the HPs from this point of view, otherwise we would have HPs who are much more sterile and much more unhappy in their doubts. (c.16.36).

#### Ways and meanings of the BU’s collaborations

##### How and why to access the BU

The participants described as ‘spontaneous’, informal (c.1.6), and personal the access to the BU (c.1.4). Sometimes, access was mediated by other professionals or directors. The two reasons most often given for contacting the BU were the need for further knowledge on Law no. 219 on Advance Directives and Advance Care planning and the wish for support to increase the quality of care as professionals (C15.10). Most of the participants reported that after the first contact with the BU they discussed and agreed with the BU’s Head which type of service would help among research activity, ethics support and ethics training. Often, the presence of the BU in the hospital has been cited as a reason to work with it (C.6.6). Some defined their collaboration with the BU as ‘*germinal*’ and ‘in progress’ (C.11.4), while others depicted their collaboration as *‘extensive’* (C.9.4*), daily* (C.10.11), and *varied* (C.15.1). Finally, a few participants reported an abrupt interruption of the collaboration due to the COVID-19 pandemic (C.13.14).

##### The BU’s role and activities

Participants involved in some of the research projects promoted by the BU defined research issues as ‘*themes [typically] involving the inner aspects of the patients and their possibility of choice*’ (C.15.3.1). Participants also appreciated the interconnection between BU activities, which led to a practical, less theoretical approach to the topics (C8.16). This was particularly noted for training and case discussions among the care team, but HPs also highlighted the novelty of the issues treated in the educational programs. (c.2). Moreover, Individual ethics consultations were requested by the HPs for reassurance and to find someone able to help them in reading difficult emotions cognitively (c.5), while ethical supervision was perceived as a more structured, multi-professional meeting among the care teams (c.11.1.5).

#### The role of the bioethicist and organizational aspects

##### Bioethicist’s personal attitudes and specific competences

The bioethicist was perceived by participants as a specialist to be involved, like other professionals, in critical case discussions. Participants appreciated different characteristics related to the personal attitude of the bioethicist, such as speed (c.7), availability, communicative competence (c.11.11), and the practical approach (c.10). One participant also noted the bioethicist’s ability to support HPs in expressing their thoughts without being judged. One participant underlined that it is essential to have someone who can help with the HPs’ moral distress. Participants identified the bioethicist as a professional with specific, advanced competences that can help them develop a different perspective in order to foster a more patient-centered decision-making process. (c.18.20;)

##### Organizational aspects

Participants generally did not identify criticisms or problems in their collaboration with the BU. However, some ‘organizational’ limits were specified. It was noted that activating the ethics consultation was difficult, especially in urgent situations due to the time constraints. In such situations, asking for ethics consultation is “already too late”, while a more proactive approach would have benefitted the HPs, preventing the ethical conflict (c.3.28). Other emerging relevant limits included the lack of other competent personnel to support the bioethicist (c.11.45) and the lack of sufficient information about the BU service and its activities (c.5.43). A few participants referred to bioethics as ‘something niche’, calling for more involvement of a bottom-up approach, for example, more intense participation of the BU personnel in the daily health care activities (c.D).

#### Impact on the HPs’ attitudes

##### A deeper way of thinking

Participants reported that the collaboration with the BU, especially in ethics consultation and research projects, led them to develop a more mature and deeper way of thinking (c.1.10), stimulating new questions and focusing on the practical and daily aspects of ethics in clinical practice (c.6). One participant specified that working with the BU helped the professionals to be more aware of the ‘limits’ of their own competences (c.7). Many others reported that it helped them to develop a holistic understanding of what is going on in a clinical case (c.13.22; c.14).

##### Identifying new questions in clinical practice

According to participants, the research activity proposed by the BU allows them to focus on the ethical aspects of health careissues. For example, only after a 4 h ethical training, pediatric HPs asked for a research project on pediatric advance care planning. Pediatric HPs interviewed also noted how the ethics support helped them better synchronize the time required by pediatric patients’ end of life and the time needed by the parents to deal with the complex situation (c.18.9; c.18.10). Many participants reported feeling *supported* (c. 5.21), *peaceful, relieved* (5.27, c.15.27), and *safe* (c.10) in making difficult decisions, in both patient-clinician relationships and interpersonal relationships among colleagues. (14.20, c. 15. 33).

#### Further needs

Participants suggested a more structured method for collaboration with the BU, starting with better identification of formal access paths (c.1.6). They also requested increasing the cooperation with a more robust integration between the BU and the operative wards, and a more proactive attitude by the BU (c.4; c.6). Participants highlighted the need to broaden a ‘common ground’ related to ethical issues in clinical practice among HPs, considering the multi-professional relevance of such activity, by taking a practical formative approach (c.7, c.13.38, c.16.36). Generally, participants called for a normalization of the ethical discussions within their clinical practice (c.6.). According to participants, end-of-life issues, communication, advance directives, research ethics, advance care planning, shared decision-making process, and ethical aspects related to COVID-19 need further in-depth analysis*.*

## Discussion

Quantitative data collected and qualitative interviews with HPs were crucial to evaluate the BU impact on clinical practice.

As quantitative results showed, the number of BU research projects increased extensively over the years and the number of work collaborations with other units and wards. We can define this process as an “inter-related growth”. Indeed, our results have shown that performing both empirical bioethics research and more traditional clinical ethics activities at the same unit would produce a mutual improvement and thus lead to synergy effects. For example, many research projects started as a result of ethics consultation, or the latter came inside a specific research activity. HPs particularly appreciated the integration of research activity, training, and EC. They stressed the need to increase such activities and spread the BU's knowledge among colleagues within the health care facilities. It represents an exciting and novel result, yet unexplored by literature—the few described experiences focused on the research activity only [24, 25] or the ethics consultation function [3, 10, 26].

According to our quantitative data, research activities lead to the BU being considered a cross-institutional service, which can be applied in different settings and with different HPs. Moreover, dealing with ethical problems through research activities would enhance mutual collaboration between ethicists and HPs as reported in the qualitative interviews. It also helps ethicists develop ethics tools and interventions based on the specific needs in the setting where the collaboration will be implemented and helps HPs consider ethics as a part of daily clinical practice. This approach is in line with others in the field of empirical bioethics, such as the 'deliberative engagement', the 'embedded researcher', and the 'committed researcher' approaches [27]. These research methods integrate a patient-oriented service and a multidisciplinary research team to gain a deeper understanding of what the different stakeholders express, and to establish ‘*a coherent and viable framework to regulate the unfolding practice, in a way that can best honor the essential life-building values of all the concerned people*' [27].

Despite the recent increasing interest and improvements in the role of empirical research in bioethics [24, 28], many challenges still affect its implementation, including the lack of training of bioethics specialists in empirical methods and the lack of consensus on the appropriate methodology for doing empirical research in bioethics [29]. A standard methodology for empirical research in bioethics is still an open and debated question [30], while our results could highlight the substantial contribution of an integrated CESS that promotes research projects along with ethics consultation.

Another important finding of this study was about the role of ethics support. According to our results, the BU ethics consultation activity over the years was focused on HPs only. The topic of patient participation in clinical ethics support service (CESS) is a matter of ongoing controversy in Europe [31]. It is increasingly discussed in the literature due to the lack of extensive theoretical or empirical studies on the matter. CESSs and clinicians have reported a positive experience regarding the participation of patients or next of kin in the consultations. It can help them understand new and important information for achieving appropriate deliberation, even though the presence of stakeholders can also bring further tension and conflict to the meetings. It is also sometimes perceived as inhibiting frank discussions [32].

As our qualitative findings highlighted, HPs appreciated the practical/pragmatic aspect of ethics education and training, describing the impact on their clinical practice in terms of the development of new questions about daily clinical practice and the meaning of 'professional competences'. Previous studies described the impact of such ethical interventions on HPs and the related outcomes [33]. These studies demonstrated that multidimensional educational intervention, combining different elements such as lectures, small-group discussions, workshops, and ethical roundtables focusing on different areas of ethics increased HPs' ethical knowledge and ethical sensitivity, the application of ethical principles and ethical codes, and the identification of ethical problems within their clinical practice [33–35].

Our qualitative findings also highlighted the supportive role of the bioethicist within the health care team. HPs felt reassured and more confident in dealing with complicated decision-making processes. These findings confirm the role of the ethicist, as described in the literature, in helping to create the appropriate environment in which ethical reflection and deliberation can take place [36].

However, the support of a single ethicist within the health care team, providing ethics consultation, represents one form of CESS. Different forms of CESSs have been developed and implemented worldwide, including the clinical ethics committee (CEC) model, mainly widespread in Europe.

A CEC is a multidisciplinary institutional body assigned to consider, debate, take action on, or report ethical issues in patient care [3]. It focuses on providing advice or recommendations to HPs concerning the best course of action in a specific clinical case or discussion. It leads to a good decision-making process through a written, institutional response. Rather than the single bioethicist, the support of a multidisciplinary and pluralistic body would be constructive in dealing with complex cases where different moral views conflict [37]. Since our results showed HPs have been progressively sensitized to their clinical practice’s ethical aspect of care, we hypothesised that they could also benefit from the support of a multidisciplinary institutional body in managing complex moral situations. Therefore, the BU promoted an empirical bioethics research project on a CEC implementation and evaluation to continue integrating different activities.

## Strengths and limitations

Our study evaluates a BU activity by integrating a quantitative and qualitative assessment. No other experience like our BU has been previously described and evaluated concerning ethics consultation, training, and empirical bioethics research. Moreover, research activity is a distinctive feature of our BU.

However, our study presents some limitations. First, this study was developed in a local context, using a convenience sample. Moreover, since no other experiences like our BU exist in Italy, our findings are limited to a single research hospital in northern Italy. No other significant quantitative data are available to compare research from BUs nationally and internationally.

## Conclusions

Acknowledging that our ultimate goal is to provide evidence of the BU activities impact on clinical practice, we believe that our results provide new knowledge on the integration of empirical research activities, ethics consultation and ethics training.

Integrating research activities provided an impetus to increase collaboration and spread an 'ethical culture' among local HPs. We can argue that such integration of activities could be a model also for other large hospitals. Further studies are needed to understand the feasibility of the model proposed in different countries and organizations.

## Data Availability

All data generated or analysed during this study are included in this published article.

## Abbreviations

BU: Bioethics Unit
HPs: Healthare Professionals
CEC: Clinical Ethics Committee
CESS: Clinical Ethics Support Services
ORH: Oncological Research Hospital
OECI: Comprehensive Clinical Cancer Institute
CNB: National Committee for bioethics

## References

[1] Aulisio MP, Arnold RM, Youngner SJ, Hudson B (2000). Health care ethics consultation: nature, goals, and competencies. A position paper from the society for health and human values-society for bioethics consultation task force on standards for bioethics consultation. Ann Intern Med.

[2] Boniolo G, Sanchini V (2017). Consulenza etica e decision-making clinic. Per comprendere e agire in epoca di medicina personalizzata.

[3] Rasoal D, Skovdahl K, Gifford M, Kihlgren A (2017). Clinical ethics support for healthcare personnel: an integrative literature review. HEC Forum.

[4] Fletcher JC (1996). What are the goals of ethics consultation? A consensus statement. J Clin Ethics.

[5] Goldenberg MJ (2005). Evidence-based ethics? On evidence-based practice and the "empirical turn" from normative bioethics. BMC Med Ethics.

[6] Borry P, Schotsmans P, Dierickx K (2005). The birth of the empirical turn in bioethics. Bioethics.

[7] De Panfilis L, Merlo DF, Satolli R, Perin M, Ghirotto L, Costantini M (2019). Clinical ethics consultation among Italian ethics committee: a mixed method study. PLoS ONE.

[8] De Panfilis L, Merlo DF, Satolli R, Coppola T, Ghirotto L, Costantini M (2018). Clinical ethics consultation and research ethics consultation: a call for Italy. Am J Bioeth.

[9] Leuter C, Petrucci C, Caponnetto V, La Cerra C, Lancia L (2018). Need for ethics support in clinical practice and suggestion for an Ethics Consultation Service: views of Nurses and Physicians working in Italian Healthcare Institutions. Ann Ist Super Sanita.

[10] Furlan E, Viafora C, Oprandi N, Cipolletta S (2019). Creare e coordinare una rete regionale di comitati etici per la pratica clinica. Risultati e lezioni apprese da uno studio qualitativo svolto in Veneto/Establishing and coordinating a regional network of healthcare ethics committees. Findings and lessons learnt from a qualitative research in the Veneto Region (Italy). Medicina E Morale.

[11] DELIBERAZIONE DELLA GIUNTA REGIONALE n. 4049 del 22 dicembre 2004 Interventi in materia di Bioetica. Istituzionalizzazione del Comitato regionale per la Bioetica. Linee-guida per la costituzione ed il funzionamento dei Comitati etici per la sperimentazione. Linee-guida per la costituzione ed il funzionamento dei Comitati etici per la pratica clinica. https://bur.regione.veneto.it/BurvServices/Pubblica/DettaglioDgr.aspx?id=178000. Accessed 8 Oct 2022.

[12] Deliberazione della Giunta regionale, Toscana. http://www301.regione.toscana.it/bancadati/atti/Contenuto.xml?id=5309988&nomeFile=Delibera_n.1219_del_22-11-2021

[13] AZIENDA SANITARIA UNIVERSITARIA INTEGRATA DI TRIESTE, Formalizzazione dell'organizzazione supportante l'etica clinica nell'A.S.U.I. di Trieste, in attuazione dell'Atto Aziendale adottato con decreto n. 476/2017. https://asugi.sanita.fvg.it/export/sites/aas1/it/documenti/all_dss/mat_info/dss_nepc_dcr_606_2017_org_etica_cl_asuits.pdf. Accessed 8 Oct 2022.

[14] National Committee of Bioethics, Clinical Ethics Committees, 31 march 2017. https://bioetica.governo.it/en/opinions/opinions-responses/clinical-ethics-committees.

[15] National Committee of Bioethics, The role of Bioethics Experts in Ethics Committees, 28 May 2021. https://bioetica.governo.it/en/opinions/opinions-responses/the-role-of-bioethics-experts-in-ethics-committees/.

[16] Petrini C (2020). Will medically-assisted suicide mean the rebirth of (clinical) ethics committees in Italy?. Med Leg J.

[17] Huxtable R, Ives J (2019). Mapping, framing, shaping: a framework for empirical bioethics research projects. BMC Med Ethics.

[18] Molewijk B, Stiggelbout AM, Otten W, Dupuis HM, Kievit J (2004). Empirical data and moral theory. A plea for integrated empirical ethics. Med Health Care Philos.

[19] Hurst S (2010). What 'empirical turn in bioethics'?. Bioethics.

[20] Website of the CEC. https://www.ausl.re.it/comitato-per-letica-nella-clinica-cec. Accessed 8 Oct 2022.

[21] Haltaufderheide J, Nadolny S, Gysels M, Bausewein C, Vollmann J, Schildmann J (2020). Outcomes of clinical ethics support near the end of life: a systematic review. Nurs Ethics.

[22] Cresswell JW, Plano-Clark VL (2010). Designing and conducting mixed method research.

[23] Gale NK, Heath G, Cameron E, Rashid S, Redwood S (2013). Using the framework method for the analysis of qualitative data in multi-disciplinary health research. BMC Med Res Methodol.

[24] Wangmo T, Provoost V (2017). The use of empirical research in bioethics: a survey of researchers in twelve European countries. BMC Med Ethics.

[25] Chadwick R, Wilson D (2018). The emergence and development of bioethics in the UK. Med Law Rev.

[26] Fox E, Tarzian AJ, Danis M, Duke CC (2022). Ethics consultation in U.S. hospitals: opinions of ethics practitioners. Am J Bioeth.

[27] Fournier V, Bretonnière S, Spranzi M (2020). Empirical research in clinical ethics: the 'committed researcher' approach. Bioethics.

[28] Ives J, Dunn M, Molewijk B, Schildmann J, Bærøe K, Frith L, Huxtable R, Landeweer E, Mertz M, Provoost V, Rid A, Salloch S, Sheehan M, Strech D, de Vries M, Widdershoven G (2018). Standards of practice in empirical bioethics research: towards a consensus. BMC Med Ethics.

[29] Davies R, Ives J, Dunn M (2015). A systematic review of empirical bioethics methodologies. BMC Med Ethics.

[30] Brooks L, Bell D (2017). Teaching, learning and assessment of medical ethics at the UK medical schools. J Med Ethics.

[31] Brierley J, Archard D, Cave E (2021). Challenging misconceptions about clinical ethics support during COVID-19 and beyond: a legal update and future considerations. J Med Ethics.

[32] Magelssen M, Pedersen R, Miljeteig I, Ervik H, Førde R (2020). Importance of systematic deliberation and stakeholder presence: a national study of clinical ethics committees. J Med Ethics.

[33] Stolt M, Leino-Kilpi H, Ruokonen M, Repo H, Suhonen R (2018). Ethics interventions for healthcare professionals and students: a systematic review. Nurs Ethics.

[34] De Panfilis L, Tanzi S, Perin M, Turola E, Artioli G (2020). "Teach for ethics in palliative care": a mixed-method evaluation of a medical ethics training programme. BMC Palliat Care.

[35] Sader J, Audétat MC, Nendaz M, Hurst S, Clavien C (2021). Design bioethics, not only as a research tool but also a pedagogical tool. Am J Bioeth.

[36] DuVal G, Clarridge B, Gensler G, Danis M (2004). A national survey of U.S. internists' experiences with ethical dilemmas and ethics consultation. J Gen Intern Med.

[37] Crico C, Sanchini V, Casali PG, Pravettoni G (2021). Evaluating the effectiveness of clinical ethics committees: a systematic review. Med Health Care Philos.

